# Cross-species chromosome painting offers new insights into the phylogenetic relationships among 16 representative species of Ipomoeeae

**DOI:** 10.3389/fpls.2025.1610698

**Published:** 2025-06-26

**Authors:** Jianying Sun, Lei Chen, Jian Sun, Zongyun Li, Yonghua Han

**Affiliations:** School of Life Sciences, Jiangsu Normal University, Xuzhou, Jiangsu, China

**Keywords:** chromosome painting, fluorescence *in situ* hybridization, Ipomoeeae, oligonucleotide probes, phylogenetic relationship

## Abstract

**Introduction:**

Previous phylogenetic studies of Ipomoeeae species have shown inconsistent results, and latest molecular analyses have classified this tribe into two major clades (Argyreiinae and Astripomoeinae) comprising seven smaller clades. The cross-species chromosome painting (CCP) analysis can offer valuable insights into the phylogenetic relationships among species.

**Methods:**

Here, we analyzed representative species from each small clade using CCP with oligonucleotide (oligo) probes derived from chromosomes 7 (7-1/7-2) and 15 (15-1/15-2) of *Ipomoea nil* to further elucidate their phylogenetic relationships.

**Results and discussion:**

We found that each probe produced specific hybridization signals exclusively on one chromosome pair in all analyzed species, suggesting that the synteny of two chromosomes have been maintained after nearly 25 million years of divergence of these species. Despite conserved synteny, distinct chromosome painting patterns were observed among species. In all analyzed 2n=30 species from Astripomoeinae clade, probes of 7-1/7-2 and 15-1/15-2 hybridized to opposite chromosomal arms of their homologous chromosomes, respectively. By contrast, 2n=30 species from Argyreiinae showed co-localization of 7-1 and major 7-2 signals on same chromosomal arm along with weaker 7-2 signals on the opposing arm, while maintaining the 15-1 and 15-2 probes to different chromosomal arms. Notably, in all analyzed 2n=28 species from two major clades, two probes from the same chromosome showed co-localization to the same chromosomal arm, indicating that inter-chromosomal translocations or rearrangements have involved these two chromosomes. Thus, CCP analysis revealed significant cytogenetic divergence between 2n=28 and 2n=30 species that challenges existing molecular-based classifications which cluster them within the same small clade. Additionally, species relationships were further resolved through physical mapping of the 5S and 45S rDNA using fluorescence *in situ* hybridization (FISH), which revealed significant interspecific variation in rDNA distribution patterns, enabling the differentiation of most species from the same clade with indistinguishable chromosome painting patterns.

## Introduction

The morning glory family (Convolvulaceae) includes 50–60 genera and 1600–1700 species, which are circumscribed within twelve tribes (Stefanović et al., 2002). More than half of the species are included in tribe Ipomoeeae Hallier f (Manos et al., 2001; Stefanović et al., 2003), which contains *Ipomoea* and nine other genera. Morning glories have served as important model systems for diverse evolutionary and molecular genetic investigations (Miller et al., 1999; Muñoz-Rodríguez et al., 2019). Resolving their phylogenetic relationships is essential for advancing these research efforts. Phylogenetic analyses incorporating both morphological and molecular data have consistently shown that *Ipomoea* is not monophyletic, with the nine other genera of Ipomoeeae nested within it (Miller et al., 1999, 2002; Manos et al., 2001; Stefanović et al., 2002). Accordingly, the option was proposed that *Ipomoea* in its broad sense can be defined as the sole genus of Ipomoeeae based on morphological evidence (Wilkin, 1999).

Phylogenetic analyses of four plastid DNA markers classify Ipomoeeae into two principal clades: Argyreiinae and Astripomoeinae (Stefanović et al., 2003). Subsequent whole plastome sequencing further resolved Astripomoeinae into five smaller clades (Batatas, Murucoides, Pes-caprae, Quamoclit, and Cairica) and Argyreiinae into two (Pes-tigridis and Obscura) (Eserman et al., 2014). Each clade is named after its oldest constituent species identified in that study. Despite their phylogenetic distinction, no clear morphological synapomorphies separate the two major clades (Argyreiinae and Astripomoeinae) (Wilkin, 1999; Wood et al., 2020). In fact, the members of both the Astripomoeinae and Argyreiinae were often grouped together in historical classifications. For instance, *Ipomoea purpurea* (L.) Roth (Astripomoeinae) and *I. pes-tigridis* L. (Argyreiinae), which share remarkably similar gross morphology, were both classified within *Ipomoea* section *Pharbitis* (Roberty, 1952). Furthermore, the taxonomy and phylogenetic relationships for Ipomoeeae species are often incongruent in previous studies (Miller et al., 1999, 2002; Manos et al., 2001; Eserman et al., 2014). For instance, Eserman et al. (2014) recovered the Batatas, Murucoides, Pes-caprae, and Quamoclit clades as a closely related group, with Cairica positioned as the basal lineage of Astripomoeinae. In contrast, earlier studies consistently resolved Cairica and Quamoclit as sister clades (Miller et al., 1999, 2002; Manos et al., 2001), highlighting persistent phylogenetic discordance in Ipomoeeae.

Chromosomal changes have been demonstrated to serve as highly informative markers for resolving phylogenetic relationships among species (Braz et al., 2018; Meng et al., 2024). Chromosome painting (CP), using DNA probes prepared from flow-sorted or microdissected chromosomes, has been shown to be a powerful tool for tracing inter-chromosomal rearrangements in evolution among related species through cross-species chromosome painting (CCP) (Thomas et al., 1998). CCP has been widely used in animal and human cytogenetic studies, revealing that species with more similar chromosomal staining patterns generally exhibit closer phylogenetic relationships (Muller et al., 2000; Ferguson-Smith and Trifonov, 2007). However, the application of CCP in plants were not successful due to the cross-hybridization of repetitive DNA sequences in the probes that cannot be efficiently blocked (Fuchs et al., 1996).

Advances in DNA synthesis technology have made it possible to simultaneously synthesize thousands of independent oligonucleotide (oligo) fragments, providing a novel approach for developing chromosome-specific painting probes in plants (Han et al., 2015). Oligo painting probes can be designed for any plant species with a sequenced genome. Since the single-copy sequences of oligos are primarily derived from conserved gene regions, oligo probes designed from one species can be used in other plants related to the target species (Jiang, 2019). To date, oligo-based CCP has been applied in chromosome evolution studies across multiple plant genera, including *Populus* (Xin et al., 2020), *Citrus* (He et al., 2020), *Fragaria* (Qu et al., 2021), *Cucumis* (Bi et al., 2020; Zhao et al., 2021), Triticeae (Li et al., 2021; Chen et al., 2024), *Saccharum* (Yu et al., 2022), *Aegilops* (Shi et al., 2022), *Thinopyrum* (Chen et al., 2023), and *Glycyrrhiza* (Meng et al., 2024).

We have previously developed chromosome-specific oligo probes for two *I. nil* chromosomes, revealing genomic architecture and interspecific relationships among three polyploid *Ipomoea* species (Sun et al., 2022). Here, 16 representative diploid *Ipomoea* species were analyzed by CCP using these *I. nil*-derived probes to evaluate the congruence between cytogenetic data and existing molecular phylogenies of Ipomoeeae, which provides new insight into the phylogenetic relationships among these species.

## Material and methods

### Taxon sampling

The representative species were selected from all seven smaller clades in Eserman et al. (2014), including *I. trifida*, *I. setosa*, *I. amnicola*, *I. hederifolia*, *I. nil*, *I. cairica*, *I. pes-tigridis*, *I. eriocarpa*, and *I. obscura* (**Supplementary Figure S1**, species labeled with a green asterisk). When original species from their study were unavailable, we selected phylogenetic proxies following Miller et al. (1999), including *I. carnea*, *I. saintronanensis*, *I. platensis*, *I. gracilis*, *I. coccinea*, *I. hederacea*, and *I. aquatic* (**Supplementary Figure S2**, species labeled with a green asterisk). Seed materials were sourced from multiple repositories, including: (1) the USDA National Plant Germplasm System (Gainesville, Florida, USA); (2) the Xuzhou Sweet Potato Research Centre (China); (3) the South China Botanical Garden, Chinese Academy of Sciences; (4) the Germplasm Bank of Wild Species in Southwest China; and (5) commercial horticultural suppliers (**Table 1**).

**Table 1:** Plant materials used in this study.

Species	Plant ID	2n	Source
Astripomoeinae			
Batatas			
*I. setosa* Ker Gawl.	PI 686433	30	US National Plant Germplasm System
*I*. *trifida* (H.B.K.) G.Don	PI 618966	30	US National Plant Germplasm System
Murucoides			
*I. carnea* Jacq.		30	Xuzhou Sweet Potato Research Centre
*I. saintronanensis* R.W. Johnson	PI 538278	30	US National Plant Germplasm System
*I. platensis* Ker Gawl.		30	Commercial suppliers
Pes-caprae			
*I. gracilis* B.Br.	PI 538270	30	US National Plant Germplasm System
*I. amnicola* Morong.	PI553010	30	US National Plant Germplasm System
Quamoclit			
*I. hederifolia* L.	Grif 6263	28	US National Plant Germplasm System
*I. coccinea* L.		28	Commercial suppliers
*I. nil* (L.) Roth		30	Commercial suppliers
*I. hederacea* Jacq.	PI 618969	30	US National Plant Germplasm System
Cairica			
*I. cairica* (L.) Sweet		30	South China Botanical Garden, Chinese Academy of Sciences
*I. aquatica* Forssk.		30	Commercial suppliers
Argyreiinae			
Pes-tigridis			
*I. pes-tigridis* L.	PI549258	28	US National Plant Germplasm System
*I. eriocarpa* R.Br.		30	The Germplasm Bank of Wild Species in Southwest China
Obscura			
*I. obscura* (L.) Ker Gawl.		30	Commercial suppliers

### Preparation of mitotic chromosomes

Young root tips were excised and treated with 2 mM 8-hydroxyquinoline at room temperature for 2 hours, followed by thorough rinsing with distilled water. The samples were then fixed in freshly prepared Carnoy’s solution (glacial acetic acid:absolute ethanol = 1:3, v/v) for 24 hours at room temperature and subsequently stored at -20°C in 70% ethanol. Prior to enzymatic digestion, fixed root tips were washed in distilled water and incubated in an enzyme mixture (2% cellulase and 1% pectolyase) at 37°C for 2 hours. The digested root tips were carefully macerated on clean glass slides using 50% acetic acid and fine-tipped forceps, followed by flame-drying over an alcohol burner.

### rDNA probes and oligo probes

The 5S and 45S rDNA probes were prepared according to Sun et al. (2022). The sequences used to develop probes were derived from the coding region of 5S rRNA, and 5.8S, 18S and 25S rRNA from *Arabidopsis thaliana* (L.) Heynhold, respectively. The probes were synthesized by the Sangon Biotech (Shanghai) Co., Ltd. The 5S rDNA probes were 5′-end labelled with 6-carboxyfluorescein (FAM), and the 45S rDNA probes were 5′-end labelled with 6-carboxytetramethylrhodamine (TAMRA). For chromosome painting, we developed four oligo-based chromosome painting probes for *I. nil* pseudo-chromosomes 7 (7–1 and 7-2) and 15 (15–1 and 15-2), which are the shortest two pseudo-chromosomes. Each probe contained oligos that were specific to half of each pseudo-chromosome. The design, amplification and labeling of oligo probes followed our published protocols (Han et al., 2015; Sun et al., 2022).

### Fluorescence *in situ* hybridization

The FISH procedure was performed according to Sun et al. (2022). Biotin-labeled probes (7–1 and 15-1) were detected using Alexa Fluor 488 streptavidin (Invitrogen), and digoxigenin-labeled probes (7–2 and 15-2) were detected using anti-digoxigenin rhodamine (Roche Diagnostics, Indianapolis, Indiana). The chromosomes were counterstained with 4,6-diamidino-2-phenylindole (DAPI) in a VectaShield antifade solution (Vector Laboratories). FISH images were captured digitally using a Leica DM6000 B fully automated upright microscope system. Gray-scale images were captured for each color channel and then merged, and final image adjustments were performed using Adobe Photoshop (Adobe Systems). For each species, at least five well-spread mitotic metaphase cells were analyzed.

## Results

### Chromosome counting and CCP analysis in 16 *Ipomoea* species

**Figure 1:**
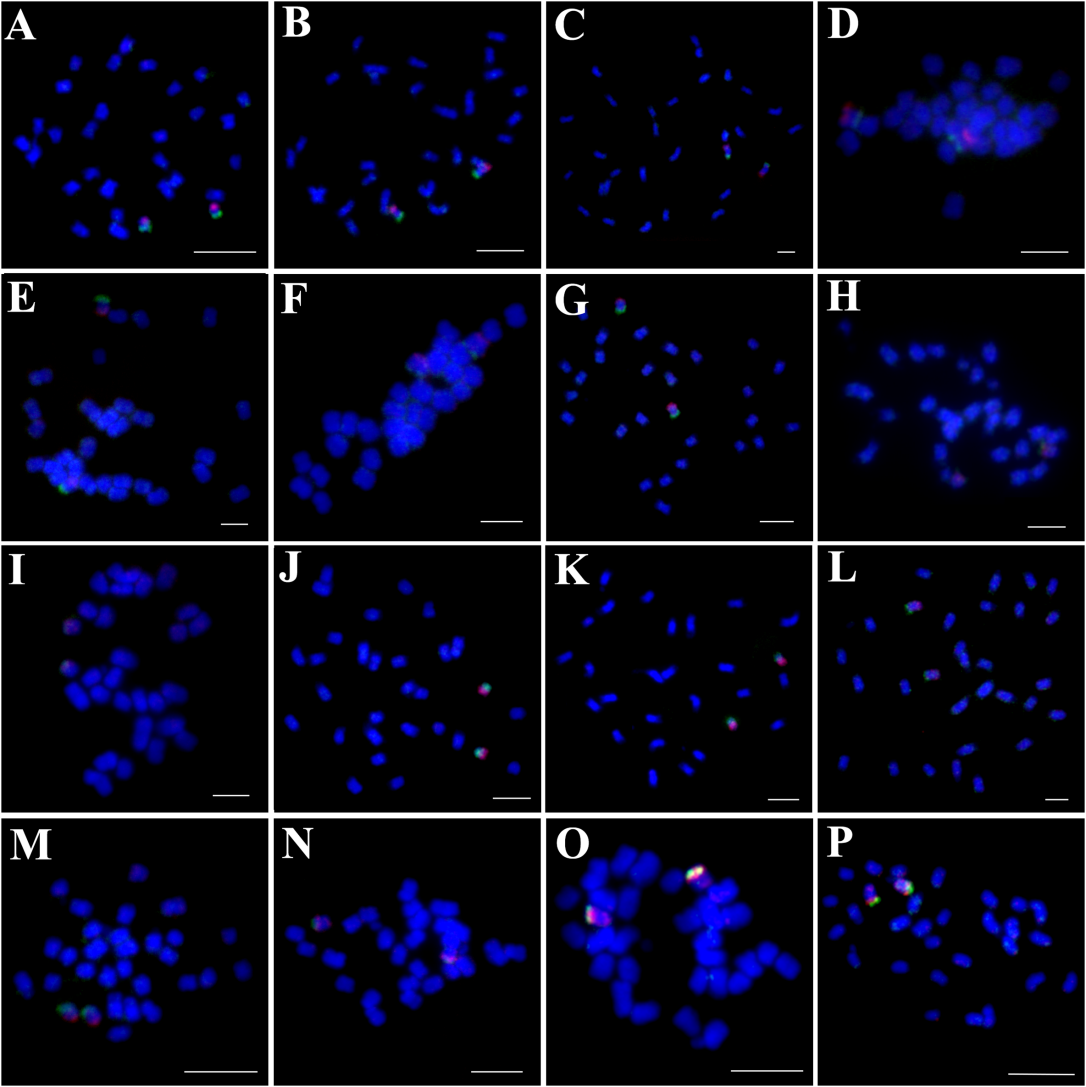
Simultaneous hybridization of oligo probes 7-1 (green) and 7-2 (red) on mitotic metaphase cell of *I*. *trifida* **(A)**, *I*. *setosa* **(B)**, *I. carnea* **(C)**, *I*. *saintronanensis* **(D)**, *I*. *platensis* **(E)**, *I. gracilis* **(F)**, *I*. *amnicola* **(G)**, *I*. *hederifolia* **(H)**, *I*. *coccinea* **(I)**, *I*. *nil* **(J)**, *I*. *hederacea* **(K)**, *I*. *cairica* **(L)**, *I*. *aquatic* **(M)**, *I. pes-tigridis* **(N)**, *I*. *eriocarpa* **(O)**, and *I. obscura* **(P)**, respectively. Scale bars = 5 μm.

**Figure 2:**
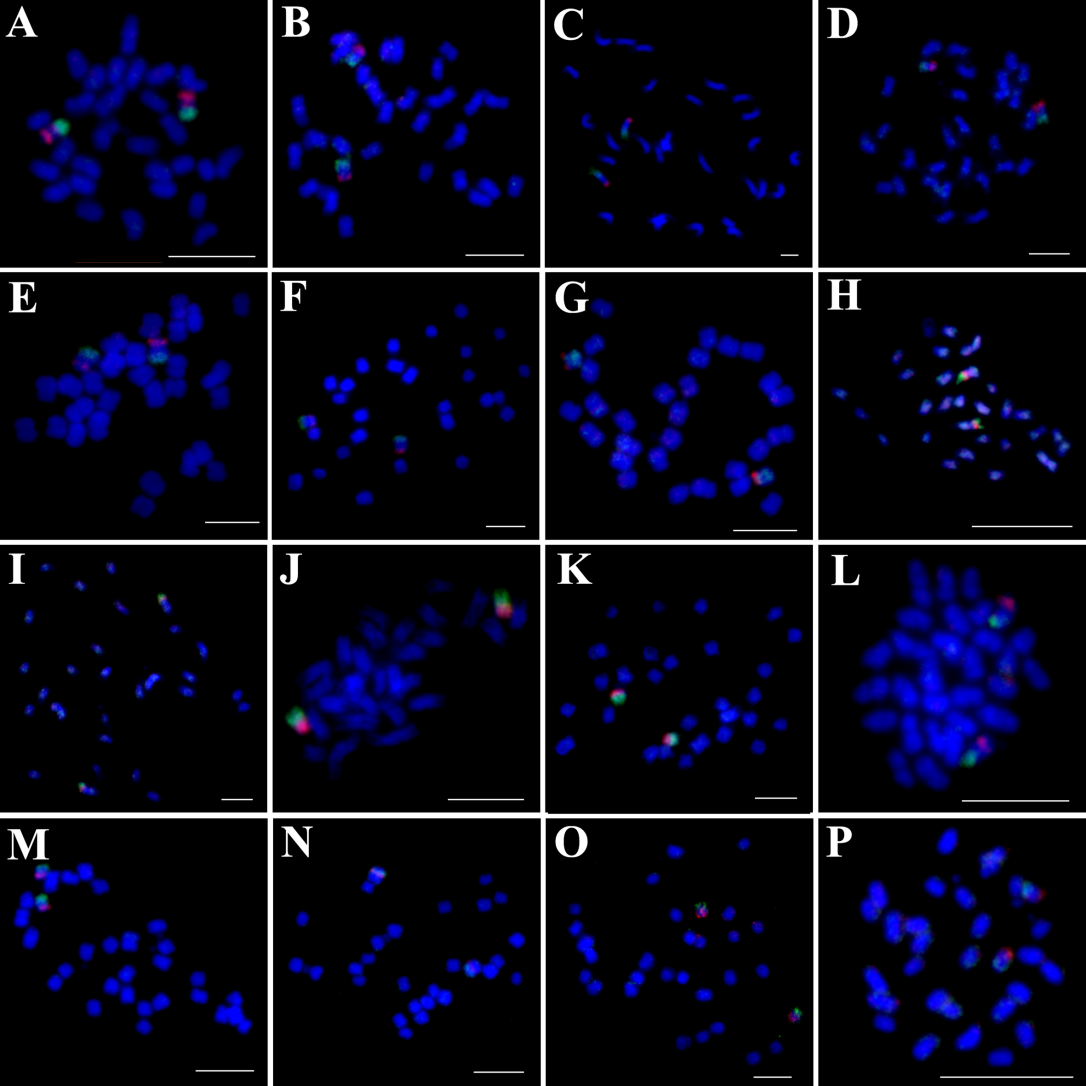
Simultaneous hybridization of oligo probes15-1 (green) and 15-2 (red) on mitotic metaphase cell of *I*. *trifida* **(A)**, *I*. *setosa* **(B)**, *I. carnea* **(C)**, *I*. *saintronanensis* **(D)**, *I*. *platensis* **(E)**, *I*. *gracilis* **(F)**, *I*. *amnicola* **(G)**, *I*. *hederifolia* **(H)**, *I*. *coccinea* **(I)**, *I*. *nil* **(J)**, *I*. *hederacea* **(K)**, *I*. *cairica* **(L)**, *I. aquatic* **(M)**, *I. pes-tigridis* **(N)**, *I*. *eriocarpa* **(O)**, and *I. obscura* **(P)**, respectively. Scale bars = 5 μm.

**Figure 3:**
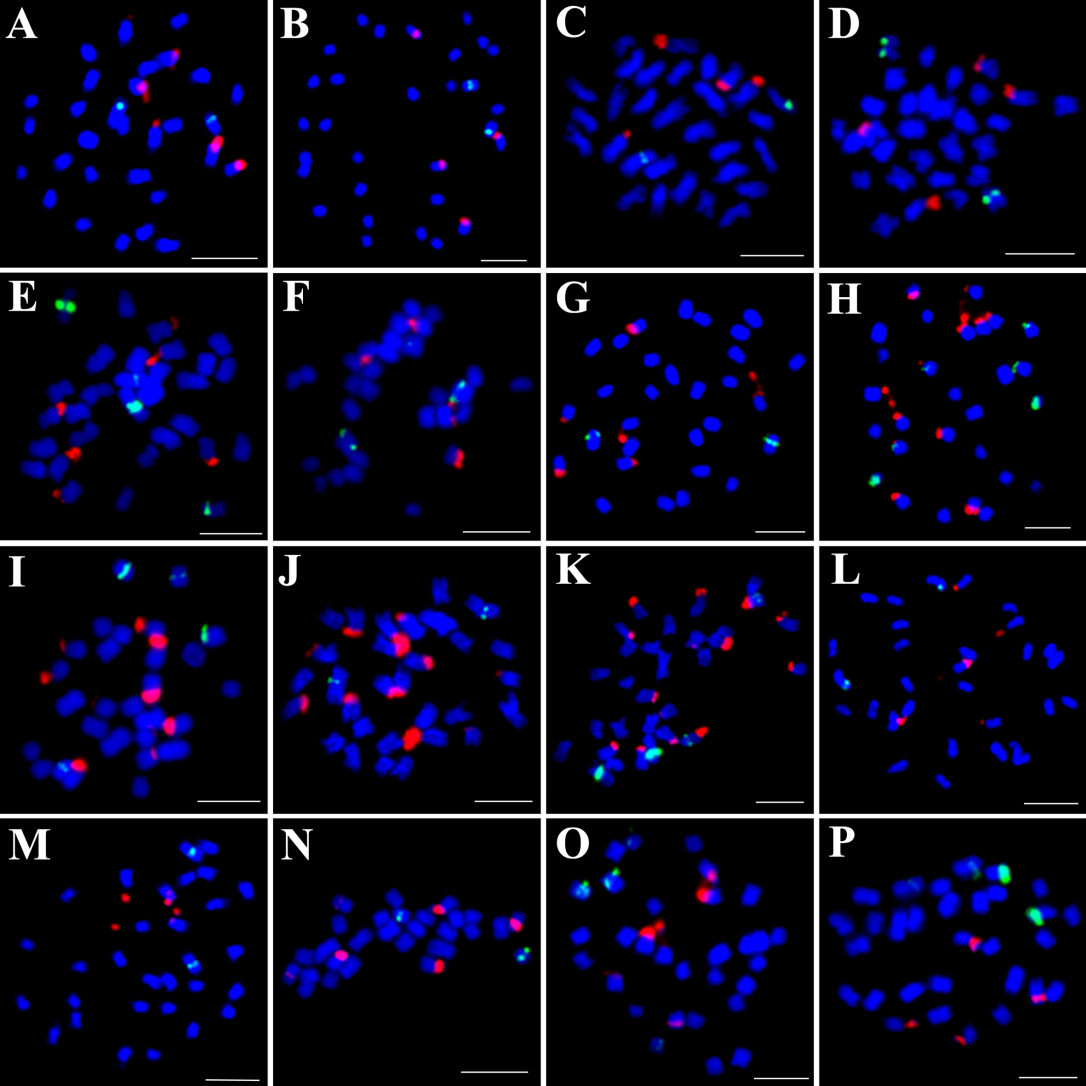
Simultaneous hybridization of 5S (green) and 45S rDNA (red) on mitotic metaphase cell of *I*. *trifida* **(A)**, *I*. *setosa* **(B)**, *I. carnea* **(C)**, *I*. *saintronanensis* **(D)**, *I*. *platensis* **(E)**, *I*. *gracilis* **(F)**, *I*. *amnicola* **(G)**, *I*. *hederifolia* **(H)**, *I*. *coccinea* **(I)**, *I*. *nil* **(J)**, *I*. *hederacea* **(K)**, *I*. *cairica* **(L)**, *I*. *aquatic* **(M)**, *I. pes-tigridis* **(N)**, *I*. *eriocarpa* **(O)**, and *I. obscura* **(P)**, respectively. Scale bars = 5 μm.

Chromosome counts revealed that 13 of the 16 examined *Ipomoea* species had 2n=30 chromosomes (**Figures 1**, **2**, **3A-G, J-M, O-P**), while *I. hederifolia*, *I. coccinea*, and *I. pes-tigridis* exhibited a reduced chromosome number of 2n=28 (**Figures 1**-**3H, I, N**), which were consistent with earlier reports for same species (Dutta, 2017; Wu et al., 2024).

**Figure 4:**
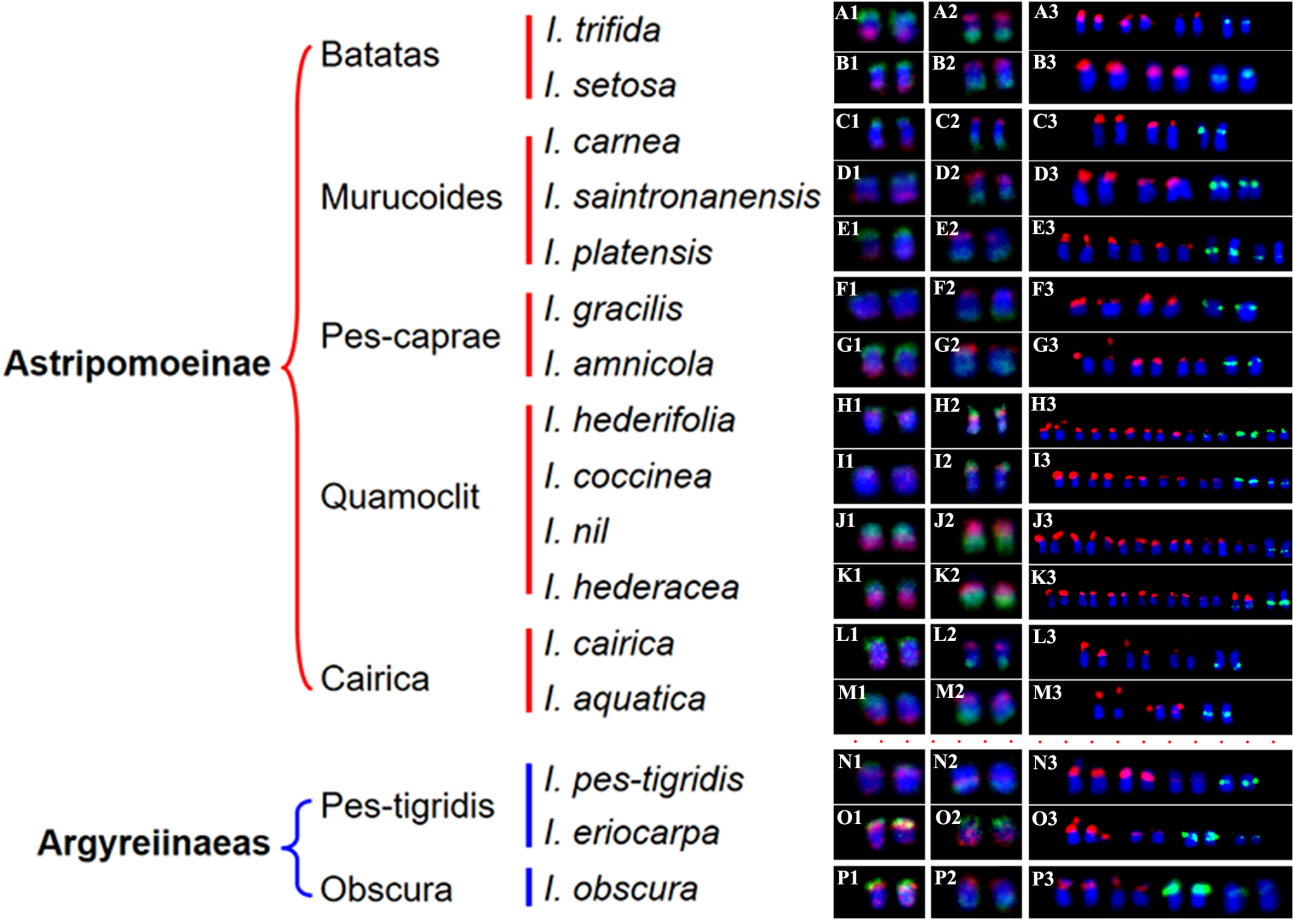
The chromosomes with FISH signals from **Figures 1**, **2**, and **3**. **(A1–P1)** show chromosomes with hybridization signals from oligo probes 7-1 (green) and 7-2 (red) in *I. trifida* **(A1)**, *I. setosa* **(B1)**, *I. carnea* **(C1)**, *I. saintronanensis* **(D1)**, *I. platensis* **(E1)**, *I. gracilis* **(F1)**, *I. amnicola* **(G1)**, *I. hederifolia* **(H1)**, *I. coccinea* **(I1)**, *I. nil* **(J1)**, *I. hederacea* **(K1)**, *I. cairica* **(L1)**, *I. aquatic* **(M1)**, *I. pes-tigridis* **(N1)**, *I. eriocarpa* **(O1)**, and *I. obscura* **(P1)**, respectively. **(A2–P2)** display chromosomes with signals from oligo probes 15-1 (green) and 15-2 (red) in the same species as A1–P1. **(A3–P3)** present chromosomes with hybridization signals from 5S rDNA (green) and 45S rDNA (red) probes in the same species as **(A1–P1)**.

The probes of 7-1/7–2 and 15-1/15–2 developed in *I. nil* were hybridized to mitotic metaphase chromosomes of these species (**Figures 1**, **2**). Each probe produced specific FISH signals exclusively on one chromosome pair, although cross-hybridization signals were also detected on other chromosomes, these signals were relatively weak and inconsistently observed. To facilitate cross-species comparison of FISH signal patterns, we digitally extracted chromosomes with FISH signals from **Figures 1**, **2** and compiled them in **Figure 4**.

In all examined species from the Batatas, Murucoides, Pes-caprae, and Cairica clades (Astripomoeinae), probes 7–1 and 7–2 exhibited specific hybridization to the telomeric regions of the long arm and short arm, respectively, leaving interstitial chromosomal regions unlabeled (**Figures 1**, **4**). In contrast, species from the Quamoclit clade displayed divergent patterns: *I. hederifolia* and *I. coccinea* (2n=28) showed complete co-localization of both probes on a single arm, while *I. nil* and *I. hederacea* displayed continuous hybridization signals along entire chromosome lengths (**Figures 1H–K**, **4**). The two Pes-tigridis clade species also exhibited distinct chromosomal painting patterns, with *I. pes-tigridis* (2n=28) (**Figure 1N**) displaying hybridization patterns identical to those observed in *I. hederifolia* and *I. coccinea* (Quamoclit clade), where both probes co-localized on a single chromosomal arm (**Figure 4**), while *I. eriocarpa* (**Figure 1O**) showed a pattern resembling *I. obscura* (Obscura clade) (**Figure 1P**) characterized by co-localized 7–1 and strong 7–2 signals on same chromosomal arm along with weaker 7–2 hybridization signals on the opposing arm (**Figure 4**).

The 15–1 and 15–2 probes exhibited hybridization patterns consistent with the 7–1 and 7–2 probes in all examined species (**Figure 4**) except *I. eriocarpa* (**Figure 2O**) and *I. obscura* (**Figure 2P**). In these two species, the 15–1 and 15–2 probes hybridized to different chromosomal arms (**Figure 4**).

### Physical localization of 5S and 45S rDNA in 16 *Ipomoea* species

To distinguish species with indistinguishable chromosome painting patterns, we conducted dual-color FISH to map the distribution patterns of 5S and 45S rDNA across 16 representative species (**Figure 3**), with signal-bearing chromosomes digitally extracted from **Figure 3** and arranged in **Figure 4** to facilitate comparative analysis. In all analyzed species except those in the Quamoclit clade, 5S rDNA loci were consistently localized to 1–2 chromosome pairs while 45S rDNA loci occupied 2–3 chromosome pairs (**Figures 3**, **4**). The Quamoclit clade species, unlike other clades, consistently exhibited higher 45S rDNA locus numbers while preserving similar 5S rDNA locus numbers relative to species in other clades (**Figures 3H–K**). Specifically, *I. hederifolia* displayed 45S rDNA signals on six chromosome pairs and 5S rDNA on three pairs, including one syntenic 5S-45S rDNA pair (**Figures 3H**, **4**), while *I. coccinea* exhibited five chromosome pairs with 45S rDNA and two pairs with 5S rDNA (**Figures 3I**, **4**). Although both *I. nil* and *I. hederacea* showed 45S rDNA signals on seven chromosome pairs (**Figures 3J, K**, **4**), they differed in their 5S rDNA locus distribution, with *I. nil* displaying 5S rDNA on a single chromosome pair (**Figures 3J**, **4**) while *I. hederacea* possessed two 5S rDNA-bearing chromosome pairs, one of which was syntenic with 45S rDNA loci (**Figures 3K**, **4**).

In all examined species, the 5S rDNA occupied proximal regions on either short or long arms, whereas 45S rDNA exhibited exclusive telomeric localization on the short arms of all chromosomes. Marked variation in rDNA signal size and intensity was observed not only among non-homologous chromosomes as well as between homologous chromosomes in several species (**Figures 4A3, H3, J3, L3, O3**), indicative of substantial rDNA copy number polymorphism. This polymorphism was also observed in other species, and underlying mechanisms were analyzed in our previous study (Hu et al., 2025). Based on the distribution patterns of 5S and 45S rDNA, species from different clades still cannot be accurately identified or distinguished, while species from same clade could be distinguished except for *I. carnea* and *I. saintronanensis* in the Murucoides clade, which showed identical rDNA signal patterns (**Figure 4**).

## Discussion

Compared with DNA probes prepared from flow-sorted or microdissected chromosomes, the synthetic oligo probes designed from single copy DNA sequences have such advantages as superior resolution and versatility, customized design, labor-saving and cost-efficient, which greatly expand the application of CCP among genetically related plant species (Jiang, 2019). In this study, we found that each probe designed from two chromosomes of *I. nil* produced specific FISH signals exclusively on one chromosome pair in all analyzed species that diverged ca. 25 million years (Eserman et al., 2014), suggesting that the chromosomal synteny has been maintained among these species. Similar examples of syntenic maintenance within the same genus have been described in multiple plant genera (Xin et al., 2020; He et al., 2020; Qu et al., 2021; Chen et al., 2024; Meng et al., 2024).

Despite conserved synteny, distinct chromosome painting patterns were observed among species. In all analyzed 2n=30 species except for *I. nil* and *I. hederacea*, oligo probes exhibited specific hybridization to the terminal regions of the chromosomes, with notably weak or absent FISH signals in interstitial regions. This could be caused by sequence divergence and/or structural expansion of these chromosomal regions. In contrast, in all analyzed 2n=28 species, two probes from the same chromosome showed co-localization to the same chromosomal arm, indicating that inter-chromosomal translocations or rearrangements have involved these two chromosomes. Building upon the correlation between phylogenetic proximity and chromosomal painting pattern similarity (Muller et al., 2000), our CCP analysis revealed a clear cytogenetic divergence between species with chromosome numbers of 2n=28 and 2n=30, challenging the current molecular-based classification that groups them within the same small clade (Eserman et al., 2014).

While CCP analysis revealed significant cytogenetic divergence between 2n=28 and 2n=30 species, all examined species from the Batatas, Murucoides, Pes-caprae, and Cairica clades (Astripomoeinae) displayed indistinguishable painting patterns for the 7-1/7–2 and 15-1/15–2 probes. The number and position of the 5S and 45S rDNA loci are important species characteristics, and closely related species usually have more similar rDNA FISH patterns than those of distantly related ones (Garcia et al., 2017). Therefore, physical mapping of rDNA loci using the FISH technique is widely used to identify species and clarify phylogenetic relationships among related species (Han et al., 2018; Qu et al., 2021; Yucel et al., 2022; Hu et al., 2025). In the genus *Ipomoea*, pioneering work by Srisuwan et al. (2006) employed physical mapping of 5S and 18S rDNA to investigate the genome organization and evolution of sweetpotato (*Ipomoea batatas* (L.) Lam.) and its seven wild relatives, and Wu et al. (2024) subsequently determined genomic distribution of 18S and 5S rDNA sites across 13 *Ipomoea* species. Our previous research (Sun et al., 2024) determined the rDNA distribution in 17 *Ipomoea* species from the Batatas clade, yielding critical insights into sweetpotato’s origin. In this study, rDNA-FISH analysis revealed that 5S rDNA loci were consistently localized to 1–2 chromosome pairs while 45S rDNA loci occupied 2–3 chromosome pairs in all analyzed species except those in the Quamoclit clade. Consequently, while rDNA distribution patterns can effectively distinguish species within same clade, they cannot be utilized for distinguishing species across different clades.

Notably, previous studies indicated close relationships between *I. hederacea* and *I. nil* with difficult morphological distinction (Austin, 1975; Austin et al., 2001), we identified diagnostic cytogenetic differences: *I. nil* possesses 5S rDNA on a single chromosome pair, whereas *I. hederacea* exhibits two 5S rDNA-bearing chromosome pairs, with one syntenic to 45S rDNA loci. Therefore, the 5S rDNA distribution pattern serves as a reliable cytogenetic marker for distinguishing between these two morphologically similar species.

## Conclusions

Taken together, this study presents the first phylogenetic analysis of Ipomoeeae species utilizing the CCP technique, revealing significant cytogenetic divergence between 2n=28 and 2n=30 species that challenges existing molecular-based classifications which cluster them within the same small clade. Our study not only provides new cytogenetic insights into the phylogenetic relationships for 16 representative species but also highlights the critical need for integrating molecular and cytogenetic data to accurately resolve complex phylogenetic relationships within this tribe. Building on these findings, future research will develop chromosome-specific oligos probes for each *I. nil* chromosome to enable detailed characterization of inter-chromosomal rearrangements and karyotype evolution across Ipomoeeae species through CCP analysis.

## Data Availability

The original contributions presented in the study are included in the article/**Supplementary Material**. Further inquiries can be directed to the corresponding authors.

## Supplementary material

Supplementary Figure 1 Phylogeny of the Ipomoeeae (*Ipomoea* and nine other genera) based on whole chloroplast genome sequences (Eserman et al., 2014).

Supplementary Figure 2 Strict consensus tree for *Ipomoea* based on simultaneous analysis of ITS and waxy sequences (Miller et al., 1999).
