# Supplementary figures and images for: Cross-species chromosome painting offers new insights into the phylogenetic relationships among 16 representative species of Ipomoeeae
Source: Front Plant Sci. 2025 Jun 26;16:1610698. doi: 10.3389/fpls.2025.1610698 (PMC12240967; doi:10.3389/fpls.2025.1610698)

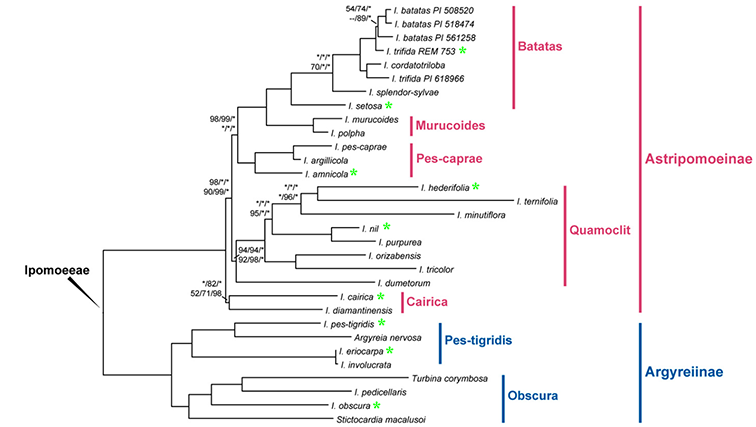

Supplement: Supplementary Figure 1 — Phylogeny of the Ipomoeeae (Ipomoea and nine other genera) based on whole chloroplast genome sequences (Eserman et al., 2014). [file Image1.tiff]

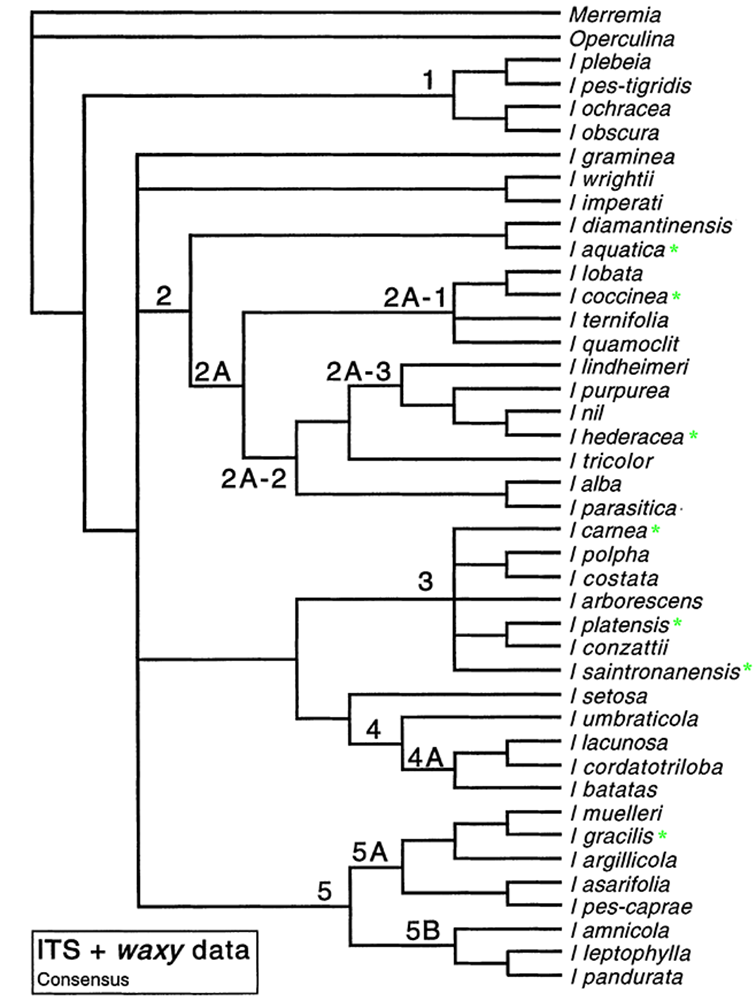

Supplement: Supplementary Figure 2 — Strict consensus tree for Ipomoea based on simultaneous analysis of ITS and waxy sequences (Miller et al., 1999). [file Image2.tif]
